# Robotic Systems for the Physiotherapy Treatment of Children with Cerebral Palsy: A Systematic Review

**DOI:** 10.3390/ijerph19095116

**Published:** 2022-04-22

**Authors:** Rocío Llamas-Ramos, Juan Luis Sánchez-González, Inés Llamas-Ramos

**Affiliations:** 1 Nursing and Physiotherapy Faculty, Universidad de Salamanca, Avda. Donantes de Sangre, s/n, 37007 Salamanca, Spain; rociollamas@usal.es (R.L.-R.); inesllamas@usal.es (I.L.-R.); 2 University Hospital of Salamanca, 37007 Salamanca, Spain

**Keywords:** robotic systems, children, cerebral palsy, physiotherapy treatments

## Abstract

Cerebral palsy is a neurological condition that is associated with multiple motor alterations and dysfunctions in children. Robotic systems are new devices that are becoming increasingly popular as a part of the treatment for cerebral palsy. A systematic review of the Pubmed, Web of Science, MEDLINE, Cochrane, Dialnet, CINAHL, Scopus, Lilacs and PEDro databases from November 2021 to February 2022 was conducted to prove the effectiveness of these devices for the treatment of motor dysfunctions in children who were diagnosed with cerebral palsy. Randomized clinical trials in Spanish and English were included. In total, 653 potential manuscripts were selected but only 7 of them met the inclusion criteria. Motor dysfunctions in the lower limbs and those that are specifically related to gait are the main parameters that are affected by cerebral palsy and the robotic systems *Lokomat*, *Innowalk*, *Robogait* and *Waltbox-K* are the most commonly used. There is no consensus about the effectiveness of these devices. However, it seems clear that they have presented a good complement to conventional physical therapies, although not a therapy as themselves. Unfortunately, the low quality of some of the randomized clinical trials that were reviewed made it difficult to establish conclusive results. More studies are needed to prove and test the extent to which these devices aid in the treatment of children with cerebral palsy.

## 1. Introduction

According to a 2007 report, “Cerebral palsy is a group of permanent disorders of the development of movement and posture, causing activity limitation, that are attributed to non-progressive disturbances that occurred in the developing fetal or infant brain” [1]. Traditionally, the diagnosis of cerebral palsy was made between 12 and 24 months of age but nowadays, diagnosis can be made before 6 months of age [2]. According to the literature, there are four possible types of cerebral palsy: spasticity (85–91%); dyskinesia (4–7%), which includes dystonia and athetosis; ataxia (4–6%); and hypotonia (2%) [2]. This pathology is the most common childhood physical disability with a prevalence of 2.1 cases per 1000 across all countries [3] and motor function disorders are the main symptoms of cerebral palsy; however, they are also often associated with other types of conditions, such as: sensation, perceptual, cognitive, communicative and behavioral disorders; epilepsy; and secondary musculoskeletal disorders. The clinical presentation of motor symptoms in cerebral palsy [4] is also very varied and there are several classifications of those symptoms. Some of the most widely used classifications are the Ingram (1955) [5] and Hagberg (1976) classifications [6].

Despite alterations or dysfunctions also occurring in the upper limbs, such as arm–hand coordination difficulties, sensory impairments and problems with grasping and manipulation tasks [7], one of the main implications of the motor impairments in children with cerebral palsy is the lower limb alterations and the difficulty with walking. It is estimated that around 90% of people with cerebral palsy have walking difficulties [8,9], specifically a decrease in walking speed and reduced endurance [10] due to their decreased cardiorespiratory fitness [11]. Therefore, the development of independent walking and gait efficiency are often the focus of therapeutic interventions for children with cerebral palsy in order to guarantee their independence in daily life [12].

There is not a gold standard for the treatment of these motor symptoms. The techniques that are commonly used to treat them are focused on early interventions that take advantage of the neuroplasticity of the brain [2]. In addition, task-specific motor training that includes self-discovery of the environment and solutions to overcome movement challenges [13,14], repeated daily practice for the acquisition and refinement of motor skills and the creation of enriched environments to promote the intensity and variety of movement, as well as enjoyable practice, are also recommended for this population [15,16]. The importance of this last aspect is very relevant since treatment that is designed as a game guarantees adherence and continuity due to the characteristics of this population. This was demonstrated by Roberts et al. [17] in their article, in which they implemented virtual reality games to assist with hand movement and function using the *Hocoma ArmeoSpring Pediatric*.

The most common treatments that have traditionally been used have tended to focus on physiotherapy and botulinum toxin [18]; however, advances in new technologies have offered up a wide range of possibilities for the treatment of this pathology. New techniques have recently appeared for the rehabilitation and treatment of the motor conditions that are caused by cerebral palsy. One of them involves a virtual reality and has demonstrated improvements in the gross motor function of the lower limbs and the fine motor function of the upper limbs in children with cerebral palsy [19]. Additionally, it is worth mentioning other new robotic system technologies, of which the *Lokomat* or *Innowalk* devices are the most significant. The objective of robotic systems is to help patients to achieve correct motor function, based on the repetition of tasks with their assistance [20]. They can provide high intensity, repetitive, task-specific and interactive training [21]. Robotics systems can focus on several strategies, such as assistive (similar to the exercises that are implemented by physiotherapists), haptic stimulation (related to the practice of daily life activities), coaching (help, motivation and promotion of motor skills learning) or challenge-based (exercises that involve more difficult tasks or challenges) [21]. The main benefits that could be obtained from the use of these devices are a decrease in spasticity and improvement in joint amplitudes, autonomy, muscle tone and strength, etc. [22,23]; however, as these are such new techniques, their feasibility and effectiveness in the treatment of this condition and within this population are not entirely clear.

As mentioned above, the *Lokomat* is one of the most widely used robotic systems, which has already been explored by van Hedel et al. [24] in their ARTIC study. This observational study included patients with neurological disorders and gait difficulties (cerebral palsy, stroke injury, spinal cord injury, traumatic brain injury, etc.) who were using the *Lokomat* system as part of their treatment in order to draw conclusions about its use and even extended its use to other diagnoses. However, no relevant conclusions have yet been obtained.

Therefore, the objectives of this study were to systematically review the evidence that is available on the effectiveness of robotic systems either as a therapy by themselves or in combination with the physiotherapy treatments of children who have been diagnosed with cerebral palsy in improving their autonomy and quality of life.

## 2. Materials and Methods

This systematic review was previously registered at PROSPERO with the ID number 308988 and the recommendations of the PRISMA statement have been followed [25].

### 2.1. Systematic Literature Research

Bibliographic research was conducted in the Pubmed, MEDLINE, Web of Science, Cochrane, PEDro, Dialnet, CINAHL, Scopus and Lilacs databases from November 2021 to February 2022. Manual bibliographic research was then performed through the references of the selected articles. The inclusion criteria were randomized clinical trials or articles, depending on the database that was being used. The language was limited to English and Spanish and the PICO strategy was followed:

**Population**: Children aged between 4 and 14 years old with a cerebral palsy diagnosis;

**Interventions**: All forms of robotic systems that assist with motor dysfunctions in addition to conventional physiotherapy;

**Comparisons**: The same group of children without the use of any robotic systems or conventional physiotherapy treatments;

**Outcomes**: The main variable to be measured was motor dysfunction, without a specific location.

### 2.2. Selection Criteria

This systematic review included randomized clinical trials with the following inclusion criteria: (1) children aged between 4 and 14 years old with a cerebral palsy diagnosis; (2) all forms and utilizations of robotic systems that are used for this population; and (3) randomized clinical trials with at least 20 patients. The exclusion criteria were: (1) a combination of treatments; (2) medical or pharmacological treatment at the same time as treatment with robotic systems or any other treatment other than robotic systems; (3) other types of publication, such as cases, editorials or letters to editors. 

### 2.3. Screening, Selection and Data Extraction

The articles that were found in all databases were reviewed and independently screened by two reviewers (L.-R.R. and S.-G.J.L.). In the first screening, duplicated articles were removed. After that, both reviewers screened the title and abstract to select eligible articles. Then, the articles were read in full. When there was any form of disagreement, a third reviewer (L.-R.I.) participated in the process in order to obtain a consensus.

Data from the fully read articles were extracted by the two reviewers (L.-R.R. and L.-R.I.). The population, age, type of robotic systems (intervention), outcome measures and follow-ups were registered in a table. When there was any form of disagreement, a third reviewer (S.-G.J.L.) participated in the process in order to obtain a consensus.

### 2.4. Assessment of Methodological Quality and Risk of Bias

To evaluate the methodological quality and the risk of bias of the selected randomized clinical trials, the physiotherapy evidence database (PEDro) scale was used. This scale was used to evaluate the sample selection, the randomization into each group, blinding (participants and therapists), initial group homogeneity and statistical analysis (intention to treat and comparisons). The total score of this scale was 10 points and a score of higher than 6 points was considered a high-quality clinical trial [26].

## 3. Results

### 3.1. Study Selection

**Figure 1:**
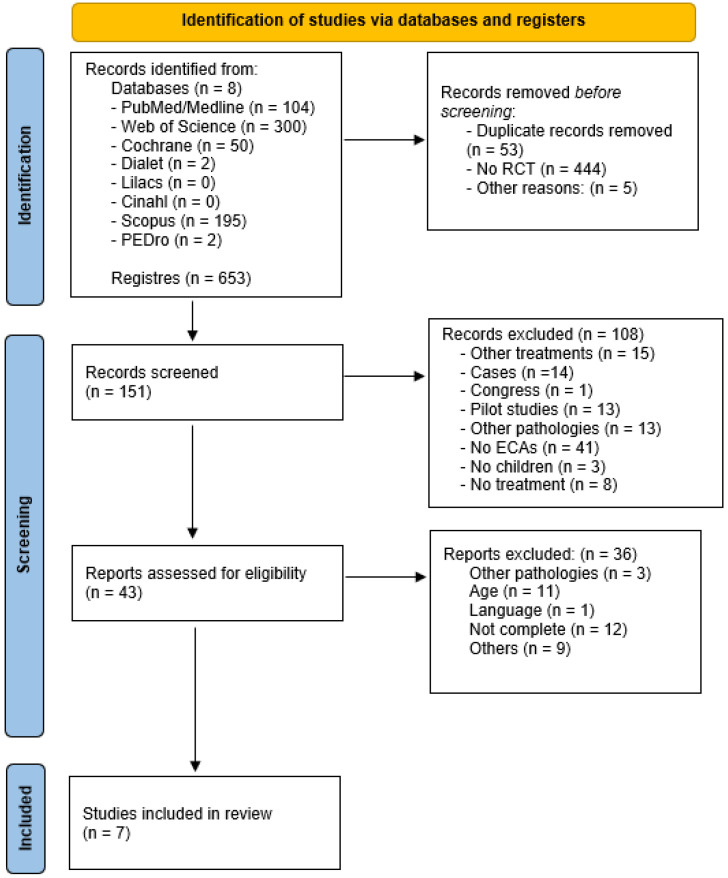
Sample selection.

In total, 653 articles were identified as potential studies for inclusion in this review. After the duplicates were removed, the sample was reduced to 151 files. The title and abstract screening then limited the sample to 43 articles, which were read in full by the two independent reviewers. Finally, the total sample that was selected following the inclusion and exclusion criteria included 7 randomized clinical trials. A PRISMA flowchart of the article selection can be seen in Figure 1.

### 3.2. Study Characteristics

The population of this systematic review was composed of children aged between 4 and 14 years old with a cerebral palsy diagnosis. Most of them received robotic system treatment as a complement to conventional physiotherapy treatment in comparison to conventional treatments only, which acted as the control group. The type of robotic system, type of treatment, treatment duration (which ranged from 4 to 12 weeks), session duration (30–40 min), main results and conclusions were extracted from each manuscript. The main characteristics of the selected articles are presented in Table 1.

### 3.3. Methodological Quality and Risk of Bias

The quality of the studies (randomized clinical trials) was assessed using the PEDro scale [26]. In our review, four articles in the sample showed high quality with 6-8 points. However, the other three articles demonstrated a low methodological quality with scores of 3, 4 and 5 points. In Table 2, the PEDro Scale assessment of the seven selected articles is presented.

### 3.4. Robotic Systems

The robotic systems that were used in the articles that were included in this systematic review mainly focused on gait parameters and lower limb motor dysfunctions. The most commonly used were the *Lokomat* system [27,28,29,32], followed by the Innowalk Pro [30], the Walkbot-K system [31] and Robogait [33].

All sessions were individualized and included between 20 [31] and 52 [27] children who were diagnosed with cerebral palsy. The duration of the sessions ranged from 25 [33] to 45 [27] min. The periodicity that was implemented in four manuscripts was 20 sessions over 4 weeks (five times a week) [27,28,29,32] and another was 18 sessions over 6 weeks (three times a week) [31]. The most diversity occurred between Yasar et al. [33], who delivered 16 sessions over 8 weeks (two sessions a week) and Yacizi et al. [30], who implement 36 sessions over 12 weeks (three times a week).

Several interventions were similar: a therapist combined with conventional physical treatments with exercises that focused on motor control, sitting stability, walking skills, stretching and strengthening exercises, squats, stair climbing and descending, functional reaching, balance and standing on a single leg, among others, with the addition of the robotic system-assisted gait training [27,30,33]. The authors established different groups, with the experimental group being the group that used the robotic systems and the control group being the group that received conventional physical treatments [28,29,32].

Despite the lack of the clear superiority of the robotic systems versus physiotherapy treatments that was found by some authors or the lack of statistically significant results between groups (*p* > 0.05) [27,33], robotic systems showed improvements in walking ability and postural and locomotor systems, which were evaluated with the Gross Motor Function Classification System, and demonstrated an improvement of 60.58% ± 14.71 in the experimental group versus 55.74% ± 15.02 in the control group after treatment for standing activities and an improvement of 50.87% ± 15.82 in the experimental group versus 43.61% ± 12.59 in the control group after treatment for walking, running and climbing activities [28,29]. Functional independence, balance and performance, which were evaluated with the Pediatric Berg Scale, showed an increase of up to 29.08 points (10.28) in the robotic system group and 26.69 points (10.82) in the control group [33]. These improvements were also reflected in walking speed (r = −0.53, *p* = 0.0011) [27] and muscle strength, which was evaluated using the lateral step-up test for the paretic limb (24.83 (5.80) in the study group versus 21.71 (4.92) in the control group), the sit to stand test (17.17 (2.37) in the study group versus 14.71 (2.75) in the control group) and the half kneeling to standing test (16.17 (3.46) in the study group versus 15.14 (2.91) in the control group). In another study, balance was again evaluated with the Pediatric Berg Scale and showed a result of 52.08 (2.68) points in the robotic group versus 51.00 (3.30) points in the control group. Additionally, the walking ability of the robotic group achieved a distance increase of 22 m, which was three times higher than the control group [30]. Functionality was measured with the Gillette Functional Assessment Questionnaire walking scale, which presented a value of 93.00 (10.11) in the study group versus 92.71 (8.88) in the control group after treatment [30]. Improvements were also observed in gross motor function and functional capability for daily activities (*p* = 0.018 for standing activities and *p* = 0.021 for dynamic activities, which were measured with the Gross Motor Function Classification System) [31]. Only one manuscript did not show any conclusive results [32]. Follow-ups are important to understand the extent or duration of the results that were obtained with the treatment. Unfortunately, only one study that was included in this review carried out a follow-up after 3 months, in which it was shown that the experimental group maintained the results that were achieved with the treatment [30]. It is important to highlight that all authors agreed that these robotic systems are a good complement to conventional physical therapy and that they cannot replace or substitute physical therapy treatments.

## 4. Discussion

New technologies and robotic systems are gaining popularity within treatment therapies and nowadays, several studies are trying to prove the feasibility and reliability of these devices in the treatment of cerebral palsy [34,35].

There is clear evidence that upper limb robotic systems improve function and strength [36], but these improvements are not always transferred to daily activities [37]. Authors have justified this by the lack of integration [38,39], as most studies have focused on the hand.

Regarding the selection criteria, only two articles were found in relation to the upper limb [17,40]. One author used the *ArmeoSpring* to prove its effectiveness in the treatment of children with cerebral palsy and acquired brain damage, which focused on the precision and velocity movement of the upper limbs [40]; the other article implemented virtual reality games to test precision movement and hand function [17]. However, neither of them were included in this review due to the exclusion criteria.

In cerebral palsy, motor impairment or the dysfunction of the lower extremities is very common [41]. There are several robotic system-assisted gait training programs that facilitate training or gait rehabilitation patterns that focus on all parameters to stimulate new rehabilitation strategies and motor function for patients who unfortunately have these alterations due to their injury. It seems that the *Lokomat* system is the most widely used, according to the reviewed bibliography [27,28,29,32]; however, no study was found that compared two different robotic systems to prove whether there is any superiority of one device over another. In this sense, Aycardi et al. [42] published an article in which they tried to verify the efficacy of the *CPWalker* platform with this same population. They highlighted the importance of investigating the gait parameters of patients with cerebral palsy. This research could be the basis for the creation of robotic systems that are adapted to the treatment of cerebral palsy, but again, there is not a clear gold standard for the selection of one device over another.

The sample presented in this systematic review consisted of 174 children and was homogeneous in terms of the population having the same diagnosis of cerebral palsy. None of the children had received surgical intervention, simultaneous treatment for cerebral palsy (e.g., muscle electrostimulation, etc.) [43] or botulinum toxin injections [18] in the last few months so that the results that were obtained with the combination of therapies would not influence the effectiveness of the robotic system that was selected by the therapists in these studies. The age ranges that were established within each manuscript varied from 2 to 9 years, whereas for Wallard et al. [28,29], the difference between the ages of the children averaged 2 years, which increased the homogeneity of their sample. Petrarca et al. and Yacizi et al. [30,32] increased this age range, which could have influenced the results as the evolution of the pathology may not be the same in a 4-year-old child as in a 13-year-old child. Petrarca et al. [32] specifically did not perform any randomization processes, but instead created two subgroups and divided the children into the groups according to age (one group for those older than 6 years and one group for those younger than 6 years), which could have produced a bias or limitation when interpreting the results due to the differences between the two study samples that were established.

The structure of the sessions and their periodicity were similar in all of the reviewed clinical trials in terms of exercises, but at the same time, they were very different. On the one hand, the therapists set up sessions three to five times a week and the duration of the treatment varied from 4 to 12 weeks, which could triple the amount of treatment from one sample to another. The duration of the sessions seemed to be uniform and averaged 30–45 min for all authors, except for Yasar et al. [33], who doubled this time to 65 min. However, it is true that a single treatment with a robotic system did not exceed 25 min. Follow-ups or post-treatment evaluations to assess the evolution or duration of the effects that were achieved with these treatments were only conducted in the short term. Only Yacizi et al. [30] stated that the results that were obtained in their experimental group (conventional therapy with *Innowalk*) were maintained after 3 months. The other authors [27,28,29,31,32,33] evaluated the immediate or short-term results, which means that more research is needed to verify the persistence of the effects that were achieved with these treatments. In addition, it should be noted that the application of these robotic systems does not involve an additional workload for the physiotherapist but it is a treatment that needs constant supervision, which is adapted to the biomechanical parameters of each child and is varied throughout its implementation according to the needs of each child [27]. On the other hand, some authors have investigated intensive treatments for these children and have concluded that exercises at home increase the amount of training, but on the downside, these exercises are less controllable [44].

Regarding effectiveness, the present review yielded contradictory evidence. Some authors showed the superior effectiveness of the use of a robotic system (*Walkbot-K*) compared to the control group after a 6-week treatment program, which achieved improvements in function and ability to perform daily activities [31]. After 12 weeks of treatment, the use of *Innowalk* also presented a clear superiority compared to the control group for Yazici et al. [30], which improved muscular fitness, balance, gait speed and peripheral O2 saturation and the results were maintained up to 3 months after the end of the treatment. Wallard et al. [28,29] demonstrated an improvement in gait in their two studies, which improved upper limb control, as well as producing better postural control and an improved gait pattern. Druzbicki et al. [27] concluded that the *Lokomat*, despite producing a slight improvement in gait, did not provide a significant improvement in the sample parameters after 4 weeks of treatment. Finally, Petrarca et al. [32] postulated that they had inconclusive results after the implementation of a 4-week treatment program with *Lokomat* and physiotherapy and that the results were not conclusive. Yasar et al. [33] stated that there was no difference between the two treatment groups, but both groups improved in functional independence, balance and gait with the established treatment. It should be noted that the short duration of the interventions is a limitation of these studies that might have altered the results and for that reason, further studies are needed to evaluate these parameters in the long term [42]. Intensive treatments have demonstrated effectiveness in motor function (in upper and lower limbs) [45] because the improvement was in relation to the amount of practice, which represents a key factor in the achievement of goals [24].

All of these devices are used in passive mode; however, a recent study that was published [46] compared the use of these devices in interactive mode for the treatment of sub-acute and chronic patients to observe cortical activation and establish comparisons between the two modes. This manuscript represents only 14 patients, but it observed that there was a change in neuroplasticity that was associated with the activation of muscle activity in the tibialis anterior during the gait, motor coordination and spasticity modifications. At the center of these studies is the method that was used by other authors [20,47]: the “assist as needed” method. This strategy helps the patients when they are not able to perform a task in order to optimize the patients’ efforts. These results open up new treatment possibilities for the recovery of these pathologies. The assistance also guarantees that the patients can progress onto more difficult tasks [21].

Most articles referenced individual treatments but Wegner and Zeaman [48] demonstrated that group treatments could benefit the patients’ learning, as well as that a treatment that is implemented by two humans could be performed faster that a human–robot team [49,50,51]. This idea justifies the interaction between patients to improve their recovery [51] through their cooperation, collaboration and competition [52].

Another characteristic that has been less investigated is unilateral or bilateral treatment. In the lower limbs, the bilaterality seems clearer but in the upper limbs, only one article was found that was specifically related to the bilateral treatment of the forearm and wrist, in which the authors stated that there is a necessity to regulate intensity treatments [53].

It is important to consider that these patients require a wide range of treatments that are not only physical but also medical and pharmacological, such as botulinum toxin [18], or the combination of other physiotherapy treatments, such as transcutaneous electrical stimulation [54], muscle electrostimulation [43], shock wave therapy [55] or kinesio taping [56], which is not only effective in the recovery of upper limb motor function but also in dysphagia disorders. There is also an article that was related to the use of music therapy for children with cerebral palsy, which showed effectiveness and the maintenance of the results for 4 months [57]. However, in this systematic review, these treatments were established as exclusion criteria to ensure that the effects or results that were obtained were due solely to the robotic systems that were used and not to the summative effects that can be achieved with a combination of several treatments.

Despite the great variability that exists in relation to the structure, periodization and duration of the sessions and treatments, most authors concluded that the addition of a robotic system into the treatment program of a child with cerebral palsy does not represent a therapy on its own, but rather that it is a good complement to the convention treatments [30]. It has also been postulated that a single treatment session with these devices (specifically with the *PeLoGAIT* device) is not effective. The authors also stressed the need for individualized treatments for each patient and for the inclusion of these devices in the holistic treatment of this population [58].

### Limitations

The main limitation that was found in this systematic review lay in the low methodological quality of some of the studies that were selected, which had no control group, no randomization when organizing the two treatment groups or a high dropout rate. Furthermore, the specificity of the sample limited the extrapolation of the results to other population groups. The inclusion of only clinical trials to establish comparisons between effective conventional treatments and the addition of robotic systems also stands out as a limitation. Despite there being no restriction in body affectation or the type of robotic system that was used, lower limb devices seem to have been more extensively investigated than upper limb, none of which were included in this review due to our exclusion criteria. Unfortunately, there are very few studies that meet our chosen criteria as most of them are isolated case studies, pilot studies or protocols that, in some cases, have not been implemented, which prevents results from being obtained.

## 5. Conclusions

The use of robotic systems is beginning to gain popularity when used in conjunction with the conventional physiotherapy treatment of children who have been diagnosed with cerebral palsy. Motor and function alterations in gait parameters have been the most commonly investigated in the literature. The *Lokomat* system seems to be the most widely used, according to the present review, but there are also other systems, such as the *Innowalk*, *Walkbot-K* and *Robogait* systems. Despite the scarce evidence that was found in the literature and the controversy that is related to the effectiveness of these systems for the treatment of children with cerebral palsy, the use of robotic systems does not represent a treatment to be applied in isolation but could be considered as an effective complement to conventional physical therapies. More studies with larger samples, better methodological quality and long-term follow-ups are needed to verify the therapeutic scope of these new technologies.

## Figures and Tables

**Table 1:** Characteristics of the selected articles.

Author (Year)	Population	Robotic System	Intervention	Results and Conclusions
**MARIUSZ DRUZBICKI** **(2013)** [27]	52 children with spastic diplegic cerebral palsy (6–13 years)	*Lokomat* active orthosis	Individual exercise program (motor control, sitting stability and walking skills) with *Lokomat* or individual exercise program; 20 sessions of 45 min	Walking speed showed a low level of improvement in both groups (r = −0.53, *p* = 0.0011). No statistically significant changes in gait parameters after 4 weeks. *Lokomat*: no significative improvement (*p* > 0.05).
**LAURA WALLARD (2017)** [28]	30 children with bilateral spastic cerebral palsy (8–10 years)	*Lokomat Pediatric*	*Lokomat Pediatric* or physical/occupational therapy treatment (passive–active mobilizations, balance, grasping and displacements); 20 sessions: 5 times perweek (40 min) for 4 weeks	*Lokomat* improved walking ability, upper body control and lower limb kinematics. Standing activity: 60.58% ± 14.71 for experimental group versus 55.74% ± 15.02 for control group. 50.87% ± 15.82 for experimental group versus 43.61% ± 12.59 for control group for walking, running and climbing activities.
**LAURA WALLARD (2018)** [29]	30 children with bilateral spastic cerebral palsy (8–10 years)	*Lokomat Pediatric*	*Lokomat Pediatric* or physical/occupational therapy treatment for walking on level ground or a mat and balance (unipodal or bipodal) up and down stairs; 20 sessions: 5 times per week (40 min) for 4 weeks	*Lokomat system* favored new gait organization to improve postural and locomotor functions and gait patterns: *p* = 0.073 (experimental group) versus *p* = 0.048 (control group). *Lokomat system* did not replace other therapies, it was a complement.
**MELTEN YAZICI** **(2019)** [30]	24 children with congenital spastic hemiparetic cerebral palsy (5–12 years)	*Innowalk Pro*: robotic gait training program	Conventional treatment (stretching and strengthening exercises, squats, stair climbing and descending, functional reaching, balance and standing on a single leg) with RGTP or conventional treatment; 3 times per week (30 min) for 12 weeks	Muscle strength, balance (52.08 (2.68) points for experimental group versus 51.00 (3.30) for control group), walking speed (22 m for experimental group), functionality (93.00 (10.11) for experimental group versus 92.71 (8.88) for control group), endurance and peripheral O2 saturation improved. Experimental group: effects were preserved after three months. Robotic system rehabilitation: supportive tool, but not a therapeutic method alone.
**LI HUA JIN** **(2020)** [31]	20 children with cerebral palsy (4–9 years)	*Walkbot-K system* (exoskeletal robot-assisted gait training)	Conventional physical therapy (2–4 times per week) or robotic system-assisted gait training (3 times per week, 30 min); 18 sessions over 6 weeks	Gross motor function and functional capability in daily activities improved after 6 weeks of treatment with the *Walkbot-K system* (*p* = 0.018 for standing activities and *p* = 0.021 for dynamic activities).
**MAURIZIO PETRARCA (2020)** [32]	24 children with diplegic cerebral palsy (4–13 years)	*Lokomat*	Robotic system-assisted gait training and physiotherapy (functional gait exercises); 20 sessions: 5 times per week for 4 weeks	Robotic system-assisted gait training with *Lokomat* and physiotherapy produced no conclusive results in the search for new adaptative solutions for these children.
**BURAK YASAR** **(2021)** [33]	26 children with diplegic cerebral palsy (7–14)	*Robogait*	Conventional physical therapy (40 min) and *RoboGait* (25 min) 2 times per week or conventional therapy alone (65 min) 2 times per week; 16 sessions over 8 weeks	No significant differences were observed between groups, although both groups showed improvement in functional independence, balance and performance at the end of the therapy (29.08 points (10.28) in robotic system group versus 26.69 points (10.82) in control group). *Robogait* alone was not superior to conventional physical therapy (*p* > 0.05).

**Table 2:** Assessment of methodological quality using the PEDro scale.

	M. DRUZBICKI (2013)	L. WALLARD (2017)	L. WALLARD (2018)	M. YACIZI (2019)	LH. JIN (2020)	M. PETRARCA (2020)	B. YASAR (2021)
**RANDOM PARTICIPANT ALLOCATION**	Y	Y	Y	N	Y	N	Y
**CONCEALED ALLOCATION**	Y	Y	Y	N	Y	N	Y
**GROUPS SIMILAR AT BASELINE**	Y	Y	Y	Y	Y	NA	Y
**SUBJECT BLINDING**	N	N	N	N	N	N	N
**THERAPIST BLINDING**	N	N	N	N	N	N	Y
**ASSESSOR BLINDING**	Y	Y	N	N	Y	N	Y
**LESS THAN 15% DROPOUT**	N	Y	N	Y	Y	Y	Y
**INTENTION TO TREAT ANALYSIS**	N	N	N	N	N	N	N
**STATISTICAL COMPARISONS BETWEEN GROUPS**	Y	Y	Y	Y	Y	Y	Y
**POINT MEASURES AND VARIABILITY DATA**	Y	Y	Y	Y	Y	Y	Y
**TOTAL SCORE**	6/10	7/10	5/10	4/10	7/10	3/10	8/10

Y, yes; N, no; NA, not applicable.

## Data Availability

All data are available under reasonable request to the corresponding author and Supplementary Materials.

## Supplementary Materials

The following supporting information can be downloaded at: https://www.mdpi.com/article/10.3390/ijerph19095116/s1.

## References

[1] Rosenbaum P., Paneth N., Leviton A., Goldstein M., Bax M., Damiano D., Dan B., Jacobsson B. (2007). A report: The definition and classification of cerebral palsy April 2006. Dev. Med. Child Neurol. Suppl..

[2] Novak I., Morgan C., Adde L., Blackman J., Boyd R.N., Brunstrom-Hernandez J., Cioni G., Damiano D., Darrah J., Eliasson A.C. (2017). Early, accurate diagnosis and early intervention in cerebral palsy: Advances in diagnosis and treatment. JAMA Pediatr..

[3] Oskoui M., Coutinho F., Dykeman J., Jetté N., Pringsheim T. (2013). An update on the prevalence of cerebral palsy: A systematic review and meta-analysis. Dev. Med. Child Neurol..

[4] Sadowska M., Sarecka-Hujar B., Kopyta I. (2020). Cerebral Palsy: Current Opinions on Definition, Epidemiology, Risk Factors, Classification and Treatment Options. Neuropsychiatr. Dis. Treat..

[5] Balf C.L., Ingram T.T.S. (1955). Problems in the Classification of Cerebral Palsy in Childhood. Br. Med. J..

[6] Hagberg G., Hagberg B., Olow I. (1976). The changing panorama of cerebral palsy in Sweden 1954—1970 III. The Importance of Foetal Deprivation of Supply. Acta Paediatr..

[7] Simon-Martinez C., Jaspers E., Mailleux L., Desloovere K., Vanrenterghem J., Ortibus E., Molenaers G., Feys H., Klingels K. (2017). Negative Influence of Motor Impairments on Upper Limb Movement Patterns in Children with Unilateral Cerebral Palsy. A Statistical Parametric Mapping Study. Front. Hum. Neurosci..

[8] Hutton J.L., Pharoah P.O.D., Rosenbloom L. (2002). Effects of cognitive, motor, and sensory disabilities on survival in cerebral palsy. Arch. Dis. Child..

[9] Pharoah P.O.D., Cooke T., Johnson M.A., King R., Mutch L. (1998). Epidemiology of cerebral palsy in England and Scotland, 1984–1989. Arch. Dis. Child. Fetal Neonatal Ed..

[10] Bjornson K.F., Zhou C., Stevenson R., Christakis D., Song K. (2014). Walking activity patterns in youth with cerebral palsy and youth developing typically. Disabil. Rehabil..

[11] Gorter H., Holty L., Rameckers E.E., Elvers H.J., Oostendorp R.A. (2009). Changes in Endurance and Walking Ability Through Functional Physical Training in Children with Cerebral Palsy. Pediatr. Phys. Ther..

[12] Wu M., Kim J., Gaebler-Spira D.J., Schmit B.D., Arora P. (2017). Robotic Resistance Treadmill Training Improves Locomotor Function in Children with Cerebral Palsy: A Randomized Controlled Pilot Study. Arch. Phys. Med. Rehabil..

[13] Kolobe A., Fagg T.H.A. (2019). Robot Reinforcement and Error-Based Movement Learning in Infants with and Without Cerebral Palsy. Phys. Ther..

[14] Dusing S.C., Harbourne R.T., Lobo M.A., Westcott-McCoy S., Bovaird J.A., Kane A.E., Syed G., Marcinowski E.C., Koziol N.A., Brown S.E. (2019). A Physical Therapy Intervention to Advance Cognitive and Motor Skills: A Single Subject Study of a Young Child with Cerebral Palsy. Pediatr. Phys. Ther..

[15] Kolb B., Harker A., Gibb R. (2017). Principles of plasticity in the developing brain. Dev. Med. Child Neurol..

[16] Nithianantharajah J., Hannan A. (2006). Enriched environments, experience-dependent plasticity and disorders of the nervous system. Nat. Rev. Neurosci..

[17] Roberts H., Shierk A., Clegg N.J., Baldwin D., Smith L., Yeatts P., Delgado M.R. (2020). Constraint Induced Movement Therapy Camp for Children with Hemiplegic Cerebral Palsy Augmented by Use of an Exoskeleton to Play Games in Virtual Reality. Phys. Occup. Ther. Pediatr..

[18] Balgayeva M., Bulekbayeva S. (2018). Effectiveness of the combined use of robotic kinesiotherapy and botulinum therapy in the complex rehabilitation of children with cerebral palsy. Asian J. Pharm. Clin. Res..

[19] Ren K., Gong X., Zhang R., Chen X. (2016). Effects of virtual reality training on limb movement in children with spastic diplegia cerebral palsy. Zhongguo Dang Dai Er Ke Za Zhi.

[20] Ivanova E., Krause A., Schalicke M., Schellhardt F., Jankowski N., Achner J., Schmidt H., Joebges M., Kruger J. (2017). Let’s do this together: Bi-Manu-Interact, a novel device for studying human haptic interactive behavior. IEEE Int. Conf. Rehabil. Robot..

[21] Marchal-Crespo L., Reinkensmeyer D.J. (2009). Review of control strategies for robotic movement training after neurologic injury. J. Neuroeng. Rehabil..

[22] Cherni Y., Girardin-Vignola G., Ballaz L., Begon M. (2019). Reliability of maximum isometric hip and knee torque measurements in children with cerebral palsy using a paediatric exoskeleton—*Lokomat*. Neurophysiol. Clin. Neurophysiol..

[23] Tornberg A.B., Lauruschkus K. (2020). Non-ambulatory children with cerebral palsy: Effects of four months of static and dynamic standing exercise on passive range of motion and spasticity in the hip. PeerJ.

[24] Van Hedel H.J., Severini G., Scarton A., O’Brien A., Reed T., Gaebler-Spira D., Egan T., Meyer-Heim A., Graser J., Chua K. (2018). Correction to: Advanced Robotic Therapy Integrated Centers (ARTIC): An international collaboration facilitating the application of rehabilitation technologies. J. Neuroeng. Rehabil..

[25] Moher D., Liberati A., Tetzlaff J., Altman D.G., PRISMA Group (2009). Preferred reporting items for systematic reviews and meta-analyses: The PRISMA statement. PLoS Med..

[26] Maher C.G., Sherrington C., Herbert R.D., Moseley A.M., Elkins M. (2003). Reliability of the PEDro Scale for Rating Quality of Randomized Controlled Trials. Phys. Ther..

[27] Drużbicki M., Rusek W., Snela S., Dudek J., Szczepanik M., Zak E., Durmala J., Czernuszenko A., Bonikowski M., Sobota G. (2013). Functional effects of robotic-assisted locomotor treadmill thearapy in children with cerebral palsy. J. Rehabil. Med..

[28] Wallard L., Dietrich G., Kerlirzin Y., Bredin J. (2017). Robotic-assisted gait training improves walking abilities in diplegic children with cerebral palsy. Eur. J. Paediatr. Neurol..

[29] Wallard L., Dietrich G., Kerlirzin Y., Bredin J. (2018). Effect of robotic-assisted gait rehabilitation on dynamic equilibrium control in the gait of children with cerebral palsy. Gait Posture.

[30] Yazıcı M., Livanelioğlu A., Gücüyener K., Tekin L., Sümer E., Yakut Y. (2019). Effects of robotic rehabilitation on walking and balance in pediatric patients with hemiparetic cerebral palsy. Gait Posture.

[31] Jin L.H., Yang S., Choi J.Y., Sohn M.K. (2020). The Effect of Robot-Assisted Gait Training on Locomotor Function and Functional Capability for Daily Activities in Children with Cerebral Palsy: A Single-Blinded, Randomized Cross-Over Trial. Brain Sci..

[32] Petrarca M., Frascarelli F., Carniel S., Colazza A., Minosse S., Tavernese E., Castelli E. (2021). Robotic-assisted locomotor treadmill therapy does not change gait pattern in children with cerebral palsy. Int. J. Rehabil. Res..

[33] Yaşar B., Atıcı E., Razaei D.A., Saldıran T. (2021). Effectiveness of Robot-Assisted Gait Training on Functional Skills in Children with Cerebral Palsy. J. Pediatr. Neurol..

[34] Fang Y., Lerner Z.F. (2021). Feasibility of Augmenting Ankle Exoskeleton Walking Performance with Step Length Biofeedback in Individuals with Cerebral Palsy. IEEE Trans. Neural Syst. Rehabil. Eng..

[35] Gerber C.N., Kunz B., Van Hedel H.J.A. (2016). Preparing a neuropediatric upper limb exergame rehabilitation system for home-use: A feasibility study. J. Neuroeng. Rehabil..

[36] Mehrholz J., Hädrich A., Platz T., Kugler J., Pohl M. (2012). Electromechanical and robot-assisted arm training for improving gene. Phys. Rev. Lett..

[37] Lo A.C., Guarino P.D., Richards L.G., Haselkorn J.K., Wittenberg G.F., Federman D.G., Ringer R.J., Wagner T., Krebs H.I., Volpe B. (2010). Robot-Assisted Therapy for Long-Term Upper-Limb Impairment after Stroke. N. Engl. J. Med..

[38] Timmermans A.A., Seelen H.A., Willmann R.D., Kingma H. (2009). Technology-assisted training of arm-hand skills in stroke: Concepts on reacquisition of motor control and therapist guidelines for rehabilitation technology design. J. Neuroeng. Rehabil..

[39] Oujamaa L., Relave I., Froger J., Mottet D., Pelissier J.-Y. (2009). Rehabilitation of arm function after stroke. Literature review. Ann. Phys. Rehabil. Med..

[40] Biffi E., Maghini C., Cairo B., Beretta E., Peri E., Altomonte D., Mazzoli D., Giacobbi M., Prati P., Merlo A. (2018). Movement Velocity and Fluidity Improve after Armeo^®^ Spring Rehabilitation in Children Affected by Acquired and Congenital Brain Diseases: An Observational Study. BioMed Res. Int..

[41] Dobson F., Morris M.E., Baker R., Wolfe R., Graham H.K. (2006). Clinician agreement on gait pattern ratings in children with spastic hemiplegia. Dev. Med. Child Neurol..

[42] Aycardi L.F., Cifuentes C.A., Múnera M., Bayón C., Ramírez O., Lerma S., Frizera A., Rocon E. (2019). Evaluation of biomechanical gait parameters of patients with Cerebral Palsy at three different levels of gait assistance using the CPWalker. J. Neuroeng. Rehabil..

[43] Pool D., Elliott C., Bear N., Taylor N., Valentine J. (2020). Locomotor training in children with cerebral palsy: Is Functional Electrical Stimulation integrated robotic training effective? A randomized controlled trial. Dev. Med. Child Neurol..

[44] Myrhaug H.T., Østensjø S., Larun L., Odgaard-Jensen J., Jahnsen R. (2014). Intensive training of motor function and functional skills among young children with cerebral palsy: A systematic review and meta-analysis. BMC Pediatr..

[45] Bleyenheuft Y., Ebner-Karestinos D., Surana B., Paradis J., Sidiropoulos A., Renders A., Friel K.M., Brandao M., Rameckers E., Gordon A. (2017). Intensive upper- and lower-extremity training for children with bilateral cerebral palsy: A quasi-randomized trial. Dev. Med. Child Neurol..

[46] Shin J., Yang S., Park C., Lee Y., You S.H. (2022). Comparative effects of passive and active mode robot-assisted gait training on brain and muscular activities in sub-acute and chronic stroke. NeuroRehabilitation.

[47] Emken J.L., Bobrow J.E., Reinkensmeyer D.J. Robotic movement training as an optimization problem: Designing a controller that assists only as needed. Proceedings of the 9th International Conference on Rehabilitation Robotics.

[48] Wegner N., Zeaman D. (1956). Team and Individual Performances on a Motor Learning Task. J. Gen. Psychol..

[49] Reed K., Peshkin M.A. (2008). Physical Collaboration of Human-Human and Human-Robot Teams. IEEE Trans. Haptics.

[50] Reed K., Peshkin M., Hartmann M.J., Grabowecky M., Patton J., Yishton P.M. (2006). Haptically linked dyads are two motor-control systems better than one?. Psychol. Sci..

[51] Ganesh G., Takagi A., Osu R., Yoshioka T., Kawato M., Burdet E. (2014). Two is better than one: Physical interactions improve motor performance in humans. Sci. Rep..

[52] Jarrassé N., Charalambous T., Burdet E. (2012). A Framework to Describe, Analyze and Generate Interactive Motor Behaviors. PLoS ONE.

[53] Hesse S., Schulte-Tigges G., Konrad M., Bardeleben A., Werner C. (2003). Robot-assisted arm trainer for the passive and active practice of bilateral forearm and wrist movements in hemiparetic subjects. Arch. Phys. Med. Rehabil..

[54] Ikoeva G.A., Nikityuk I.E., Kivoenko O.I., Moshonkina T.R., Solopova I.A., Sukhotina I.A., Vissarionov S.V., Umnov V.V., Gerasimenko Y.P. (2016). Clinical, neurological, and neurophysiological evaluation of the efficiency of motor rehabilitation in children with cerebral palsy using robotic mechanotherapy and transcutaneous electrical stimulation of the spinal cord. Pediatr. Traumatol. Orthop. Reconstr. Surg..

[55] Lin Y., Wang G., Wang B. (2018). Rehabilitation treatment of spastic cerebral palsy with radial extracorporeal shock wave therapy and rehabilitation therapy. Medicine.

[56] Ramírez J.O., de la Cruz S.P. (2017). Therapeutic effects of kinesio taping in children with cerebral palsy: A systematic review. Arch. Argent. Pediatr..

[57] Marrades-Caballero E., Santonja-Medina C.S., Sanz-Mengibar J.M., Santonja-Medina F. (2018). Neurologic music therapy in upper-limb rehabilitation in children with severe bilateral cerebral palsy: A randomized controlled trial. Eur. J. Phys. Rehabil. Med..

[58] Ammann-Reiffer C., Bastiaenen C.H., Meyer-Heim A.D., van Hedel H.J. (2020). Lessons learned from conducting a pragmatic, randomized, crossover trial on robot-assisted gait training in children with cerebral palsy (PeLoGAIT). J. Pediatr. Rehabil. Med..

